# Geospatial analysis of the influence of family doctor on colorectal cancer screening adherence

**DOI:** 10.1371/journal.pone.0222396

**Published:** 2019-10-04

**Authors:** Fabrizio Stracci, Alessio Gili, Giulia Naldini, Vincenza Gianfredi, Morena Malaspina, Basilio Passamonti, Fortunato Bianconi

**Affiliations:** 1 Department of Experimental Medicine, Public Health Section, University of Perugia, Perugia, Italy; 2 Umbria Cancer Registry, Perugia, Italy; 3 School of Specialization in Hygiene and Preventive Medicine, University of Perugia, Perugia, Italy; 4 Azienda USL Umbria 1,Laboratorio Unico di Screening, Perugia, Italy; Leibniz Institute for Prevention Research and Epidemiology BIPS, GERMANY

## Abstract

**Background:**

Despite the well-recognised relevance of screening in colorectal cancer (CRC) control, adherence to screening is often suboptimal. Improving adherence represents an important public health strategy. We investigated the influence of family doctors (FDs) as determinant of CRC screening adherence by comparing each FDs practice participation probability to that of the residents in the same geographic areas using the whole population geocoded.

**Methods:**

We used multilevel logistic regression model to investigate factors associated with CRC screening adherence, among 333,843 people at their first screening invitation. Standardized Adherence Rates (SAR) by age, gender, and socioeconomic status were calculated comparing FDs practices to the residents in the same geographic areas using geocoded target population.

**Results:**

Screening adherence increased from 41.0% (95% CI, 40.8–41.2) in 2006–2008 to 44.7% (95% CI, 44.5–44.9) in 2011–2012. Males, the most deprived and foreign-born people showed low adherence. FD practices and the percentage of foreign-born people in a practice were significant clustering factors. SAR for 145 (21.4%) FDs practices differed significantly from people living in the same areas. Predicted probabilities of adherence were 31.7% and 49.0% for FDs with low and high adherence, respectively.

**Discussion:**

*FDs* showed a direct and independent effect to the CRC screening adherence of the people living in their practice. FDs with significantly high adherence level could be the key to adherence improvement.

**Impact:**

Most deprived individuals and foreigners represent relevant targets for interventions in public health aimed to improve CRC screening adherence.

## Introduction

Colorectal cancer (CRC) is the third most frequent cancer in men and the second in women worldwide [1] and represents the fourth cancer cause of death globally [2]. Incidence is higher in more developed countries (ASR world: 36.3 among males and 23.6 among females) than in less developed regions (ASR world: 13.7 among males and 9.8 among females) [1]. Although many modifiable risk factors for colorectal cancer are well-established (e.g., high consumption of red and processed meat, obesity, smoking, etc.), primary prevention requires considerable efforts [2,3]. Indeed, the adoption of westernised diet and habits has been associated with increasing colorectal cancer incidence and mortality in Eastern Europe and in other medium to high health development index (HDI) countries [4,5]. Thus, screening assumes great relevance in colorectal cancer control, particularly where primary prevention efforts are lacking [6,7]. Italy, together with other high HDI countries, shows high incidence rates of large bowel cancer [4]. Many Italian regions started screening programs based on the fecal immunochemical test (FIT) in the middle 2000s [8]. Eligible individuals are actively invited to CRC screening; participation in the program is free of charge. Despite suboptimal CRC screening adherence (Italian average 47% in 2010–2011) [9–11], CRC FIT-based screening has already determined a significant reduction in disease specific incidence and mortality in Italy [12,13].

In Umbria, a central Italian region, organized CRC FIT-based screening started in 2006. The regional program has some specificities in the Italian CRC screening landscape. While in the other Italian regions the age span for CRC screening is 50–69 years, in Umbria the target age group includes individuals aged 50–74 years, according to international guidelines [14,15]. The Umbrian population is among the oldest in the world with a long life expectancy (e.g., life expectancy at the age of 65 in 2016 both sexes, 21.2 years, source ISTAT [16]). An outreach approach was adopted. Measures to reduce barriers and to ensure high levels of adherence have been embedded in the screening program since its introduction (*e*.*g*., mailed kit, kit returned directly by priority mail, involvement of FDs) but the corrected participation reached only 45% at the first (prevalence) time and 49% at the last study round [11]. Since adherence to CRC screening is generally low, improving participation represents an important public health strategy to fully exploit the benefits of an organized screening program [8,17,18].

We investigated the determinants of screening adherence in the regional population introducing a new geographical analysis of the geocoded population. In particular, we focused on the influence of individual factors and clustering factors corresponding to health service components (i.e., FDs, health district).

## Methods

### Study population

**Fig 1:**
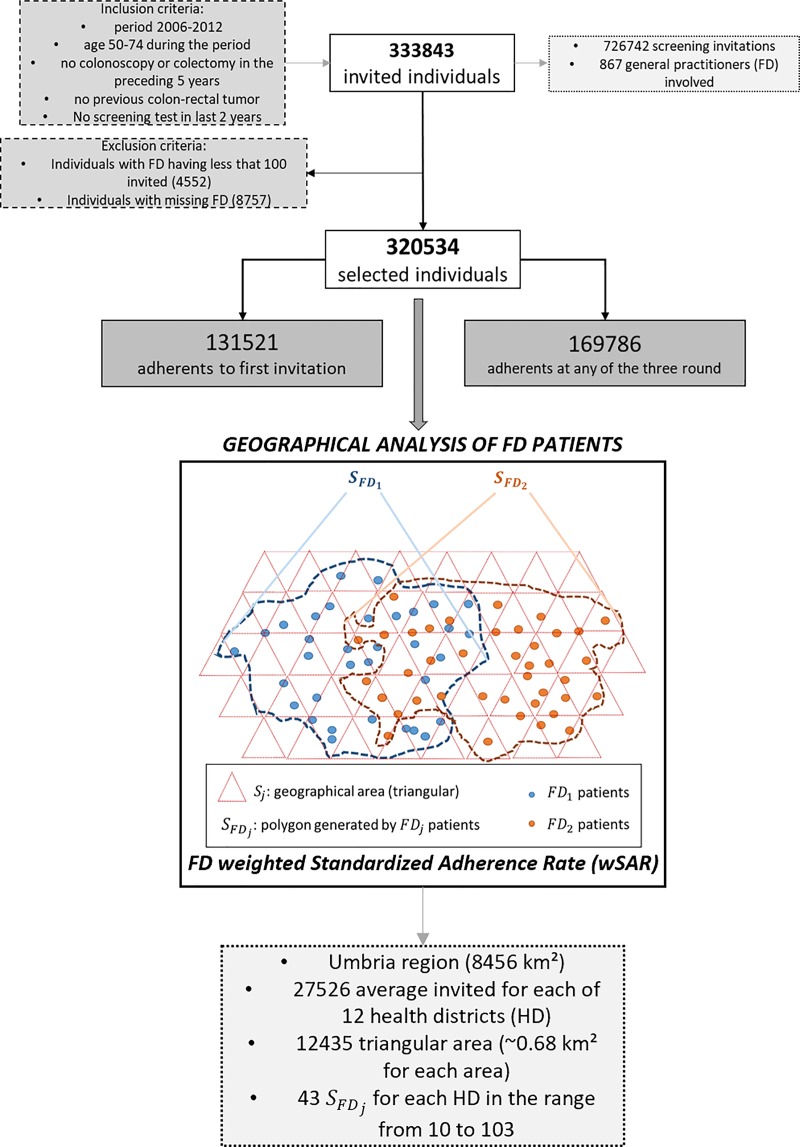
Flow diagram of study population, including a scheme of the geographical analysis used for the FD patients adherence. Figure was created by the author Bianconi F.

Data on the uptake of CRC screening were obtained from the Regional screening services. Regional prevention program was approved by Regional Government of Umbria, Management of Health and Welfare. Contact for the screening program is Dr. Basilio Passamonti, also one of the authors of this paper. Data were managed according to ISO 27001, EU General Data Protection Regulation and informed consent was obtained from all subjects included in the study. During the study period 2006–2012, overall 333,843 people aged 50–74 years were invited over three screening rounds, generating 726,742 screening invitations. Inclusion criteria were: residency in Umbria, no colonoscopy or colectomy in the preceding 5 years, no CRC screening test in the last 2 years and no personal history of CRC. In the present analyses, we considered adherence to the first screening invitation for 320,534 people (153,365 males, 47.8%). We performed further analyses on the adherence to any of the three study rounds (Fig 1).

### Study variables

We considered residence, socioeconomic status (SES), birth nationality, gender and age group as individual level determinants of CRC screening adherence. FD and health district (HD) were explored as clustering variables. Features of study clusters, such as the percentage of immigrants in a FDs practice, were also included in the models as cluster-level factors. Municipalities having less than 300 ab. over km^2^ were coded as rural. Overall, there were 15,164 (4.7%) people born abroad among the invited. The 3,909 (1.2%) people born in Western Europe were included in the Italian population, as their adherence rates were comparable to the Italian one. We considered nationality of birth as a proxy for ethnic and cultural minorities. The percentage of foreigners by cluster was also included in the analyses. SES was measured at the census tract level (micro-ecologic) using the national deprivation index (NDI). NDI is based on 5 variables (low level of education, unemployment, lack of home ownership, one parent family and overcrowding) obtained from the Italian population census (census 2001) [19] to include in a single indicator the multiple aspects of deprivation [20]. Due to incomplete information, NDI was missing for 7,166 (2.2%) invited individuals. The average number of inhabitants per census tract was 121 (min 0, max 1,475). The number of FDs was 867. FD was missing for 8,757 patients (2.6%), mainly because of recent change of residence, and was associated with a very low crude participation (11.8%). Missing FD was more prevalent among foreign-born individuals (4,483, 22.0%) than among Italians (4,274, 1.4%), reflecting the higher mobility of this population. FDs with few invited patients (i.e., < 100) were excluded from the analyses (n. 191); overall, these FDs had 4,552 (1.4%) invited patients only (Fig 1).

The areas covered by Italian local health units divide into HDs. HDs provide specialist out-patient care and other services, promote preventive activities and coordinate the FDSs activities. In Umbria, the Regional health service consists of two local health units, each including 6 HDs. The average number of invited population per district was 27,526 with a mean of 43 FDs per district (range 10–103).

### Geocoding

An extension of the Information Systems presented in [21] called GeCO-sys and based on Google Maps Geocoding API was used for geocoding and 98.7% of invited individuals were successfully geocoded. Population geocoding results were compared to age group population data from the National Institute of Statistics (ISTAT) at census level (2011). FDs were mapped using the centroid/baricentre of their patients’ addresses.

### Statistical analysis

The chi-square test assessed the impact of study variables on CRC screening participation. Results were deemed significant at the usual alpha level (0.05).

We calculated simple standardized adherence rates (SARs) by gender, age and SES. SARs over a selected area (S), i.e., triangular or hexagonal area, census section, or municipal level, were obtained using the following formula:

$${SAR}_{S}=\frac{\sum_{i}^{K}a_{S_{i}}}{\sum_{i}^{K}R_{S_{i}}I_{S_{i}}}$$

where $a_{S_{i}}$ was the number of adherence events in the i-th stratum of the study population (e.g. sex, age classes are the variables to stratify the population), $R_{S_{i}}$ was the adherence event rate in the i-th stratum of the regional standard invited population and $I_{S_{i}}$ was the size of the i-th stratum of the invited population. The smallest partition considered was the triangular area (0.68 km^2^, on average 111 invited residents, 48% males).

To investigate the role of FDs and FDs practices, we compared adherence in a FDs practice with adherence in the general population living in the same areas by local SARs. We defined the *S*_*FD*_ area as the polygon including areas (e.g., triangular areas) containing at least one FD patient (Fig 1A). The weighted FD SAR was:

$${wSAR}_{FD}\frac{\sum_{i}^{K}a_{{FD}_{i}}}{\sum_{i}^{K}\frac{a_{S_{FD_{i}}}}{I_{S_{FD_{i}}}}I_{FD_{i}}}=\frac{\sum_{i}^{K}a_{FD_{i}}}{\sum_{i}^{K}{w_{S_{FD_{i}}}I}_{FD_{i}}}$$

where $a_{FD_{i}}$ was the number of observed adherent FDs patients in the i-th stratum, $w_{S_{FD_{i}}}$ was the ratio of adherent population $a_{S_{FD_{i}}}$ over the invited population $I_{S_{FD_{i}}}$ in the area $S_{FD_{i}}$ and $I_{FD_{i}}$ was the invited FDs population. A 95% confidence interval was calculated for *SAR*_*S*_ and ${wSAR}_{S_{FD}}$.

We fitted a set of multilevel logistic regression models to investigate the influence of study variables on screening adherence [22]. Invited individuals (level 1) were considered clustering by FDs practice (level 2).

First, we fitted a random intercept empty model (i.e., without fixed effects variables) to test the influence of FDs on adherence. A second logistic regression model investigated individual-level variables as independent determinants of adherence. Then, we fitted a multi-level model including significant individual variables (fixed effects) and allowed the adherence by FDs practice to vary randomly.

The final model was selected using the Bayesian information criterion (BIC)[23]. The selected model allowed the random variation of both the intercept for FDs practices and the coefficient for the percentage of foreigners in a FDs practice and it took the following form:

$$\log(\frac{\pi_{ij}}{1-\pi_{ij}})=\beta^{\prime }x_{ij}+u_{j}^{\prime }z_{ij}$$

where the vector with fixed effects (***x***_*ij*_) was denoted by ***β*** and the vector with the random effects (***z***_*ij*_) shared by all level-1 units i, i = 1,…,*n*_*j*_, belonging to the j-th level-2 unit j, *i* = 1,…,*n*, by ***u***_*j*_. ***π***_*ij*_ = *E*(***y***_*ij*_|***x***_*ij*_,***z***_*ij*_,***u***_*j*_) was the conditional expectation of binary response ***y***_*ij*_.

Finally, we fitted two multilevel models for FDs practices respectively with significantly higher and lower local ${wSAR}_{S_{FD}}$ than the residents in the same areas.

The variance partition coefficient (VPC) was calculated as a measure of the variability explained by clustering variables (e.g., variance due to adherence levels by FDs practices or HD). In our two-level models with random intercept and random coefficient, VPC is the same as the intraclass correlation coefficient (ICC) due to a zero value for the slope variable, which is a measure of correlation among individuals belonging to the same cluster.

In a multilevel model, it is not possible to estimate the odds ratios for cluster-level variables and this poses some difficulties for the interpretation of the influence of such variables. To overcome this limitation, additional measures were proposed for cluster-level variables (reviewed in [24]). We also calculated the median odds ratio (MOR) to further illustrate the adherence heterogeneity between clusters [24]. MOR represents the median value of the odds ratio in the distribution of pairwise comparisons between subjects with equal values of covariates but belonging to different clusters. The MOR assumes values ≥1, with 1 indicating no variation among clusters. MOR is expressed in the odds ratio scale and can be properly compared to the fixed-effects odds ratios to quantify the cluster effect. The MOR can be interpreted as the (median) change in risk for an individual moving from a cluster at lower risk to another at higher risk [25].

Predicted adherence probabilities at average covariates values were calculated based on the two models above and, for comparison, using model 1, over the same selected FD practices.

We performed all analyses using Stata statistical software [26] and the GeoMap module of GeCOsys for geocoded data [21].

## Results

Adherence to the CRC screening program, excluding spontaneous participation, increased from 41.0% (95% CI, 40.8–41.2) in 2006–2008 to 44.7% (95% CI, 44.5–44.9) in 2011–2012. Among individuals invited for the first time to CRC screening, overall adherence was 40.2% (95% CI, 40.1–40.4) (S1 Fig). The distribution of participation by study variable is shown in Table 1. Adherence to at least one invitation was 53.0% (95% CI, 52.8–53.1). Low screening adherence was observed for the foreign-born, the less deprived quintile, the youngest and oldest age groups, and males. Median adherence by FDs practice was 41% and ranged from 21% to 57% (IQR 8%).

**Table 1:** Distribution of study variables by adherence to first screening invitation and adherence to any of the three study rounds.

Variables		Adherence to first screening invitation				Adherence to any of the three study rounds				
		Yes		No		Yes		No		Total
		N	%	N	%	N	%	N	%	N
Sex										
	*Female*	71,961	43.1	95,208	57.0	92,011	55	75,158	45.0	167,169
	*Male*	59,560	38.8	93,805	61.2	77,775	50.7	75,590	49.3	153,365
		p<0,001				p<0,001				
Nationality										
	*Italian*	127,054	41.6	178,316	58.4	163,776	53.6	141,594	46.4	305,370
	*Other*	4,467	29.5	10,697	70.5	6,010	39.6	9,154	60.4	15,164
		p<0,001				p<0,001				
*Socioeconomic Status (NDI)*										
	*1 Less deprived*	29,751	41.9	41,220	58.1	38,182	53.8	32,789	46.2	70,971
	*2*	24,869	42.0	34,413	58.1	32,253	54.4	27,029	45.6	59,282
	*3*	26,827	41.7	37,567	58.3	34,552	53.7	29,842	46.3	64,394
	*4*	24,067	41.0	34,579	59.0	31,023	52.9	27,623	47.1	58,646
	*5 Most deprived*	23,336	38.8	36,739	61.2	30,127	50.2	29,948	49.9	60,075
	*Missing*	2,671	37.3	4,495	62.7	3,649	50.9	3,517	49.1	7,166
		p<0,001				p<0,001				
Age										
	*50–54*	40,120	38.5	64,089	61.5	50,847	48.8	53,362	51.2	104,209
	*55–59*	23,570	41.2	33,577	58.8	32,282	56.5	24,865	43.5	57,147
	*60–64*	23,389	43.7	30,078	56.3	31,452	58.8	22,015	41.2	53,467
	*65–69*	22,791	45.0	27,857	55.0	29,591	58.4	21,057	41.6	50,648
	*70–74*	21,651	39.3	33,412	60.7	25,614	46.5	29,449	53.5	55,063
		p<0,001				p<0,001				
Round										
	*First*	93,236	41.5	131,588	58.5	124,332	55.3	100,492	44.7	224,824
	*Second*	27,010	40.0	40,491	60.0	33,968	50.3	33,533	49.7	67,501
	*Third*	11,275	40.0	16,934	60.0	11,486	40.7	16,723	59.3	28,209
		p<0,001				p<0,001				
Total		131,521	41.0	189,013	59.0	169,786	53.0	150,748	47.0	320,534

**Fig 2:**
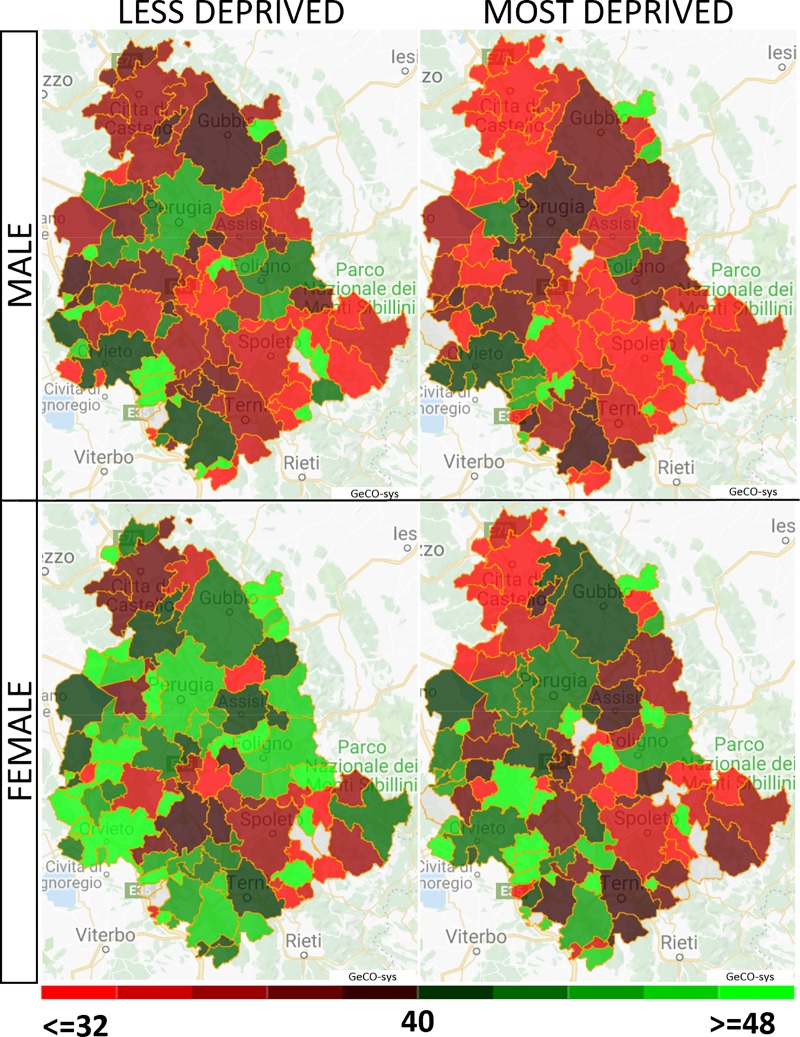
Regional screening adherence mapped as SAR_s_ by municipality, gender and extreme deprivation categories. Figure was created by the author Bianconi F. combing the caterpillar plots and maps generate with GeCO-sys an extension of [21].

The maps of standardized screening participation (*SAR*_*S*_) by municipality, gender and deprivation are shown in Fig 2. Male gender and the most deprived were associated with low CRC screening adherence.

In regression modelling, after estimating random intercept of empty model (i.e., without fixed effects variables) a significant independent effect on participation was observed for FDs practice (LR test p<0.00001), disclosing that the between FDs variance is non-zero).

Then, fixed effects for gender, age, birth nationality, round and NDI were included in the multilevel model with FDs practice as a cluster level variable. Urban/rural variable was non-significant and thus was excluded from the model. The model with the lowest BIC included the percentage of foreigners in each FDs practice as random coefficient (LR test p<0.00001 vs random intercept model only) (Table 2, model 1). The odds ratios for fixed effects remained unchanged to the second decimal place after the inclusion of foreign born people as a FDs practice factor. The VPC for random effects in model 1 was 7.8%. The MOR for the FDs effect was 1.12, similar to the OR estimated for deprivation effect.

**Table 2:** Estimated odds ratios of adherence to CRC screening program for multilevel logistic regression models: Random variation of the intercept for FDs practices and the coefficient for the percentage of foreigners for adherents (Model 1) and for ever-adherents (Model 2).

Variables		Model 1		Model 2	
		OR	95%CI	OR	95%CI
*Sex*					
	*Female*	1.21	1.19–1.22	1.22	1.2–1.23
	*Male(ref*.*)*	-	-	-	-
*Nationality*					
	*Italian*	1.67	1.60–1.75	1.68	1.63–1.79
	*Other(ref*.*)*	-	-	-	-
*Socioeconomic Status (NDI)*					
	*1 Less deprived*	1.13	1.10–1.16	1.13	1.11–1.16
	*2*	1.15	1.12–1.18	1.16	1.14–1.19
	*3*	1.12	1.09–1.14	1.13	1.10–1.16
	*4*	1.10	1.07–1.12	1.11	1.08–1.13
	*5 Most deprived(ref*.*)*	-	-	-	-
*Age*					
	*50–54 (ref*.*)*	-	-	-	-
	*55–59*	1.11	1.09–1.13	1.35	1.33–1.38
	*60–64*	1.22	1.20–1.25	1.47	1.44–1.51
	*65–69*	1.27	1.24–1.30	1.44	1.41–1.47
	*70–74*	1.00	0.98–1.03	0.88	0.86–0.90
*Round*					
	*First (ref*.*)*	-	-	-	-
	*Second*	1.09	1.07–1.12	-	-
	*Third*	1.15	1.11–1.18	-	-
*N*			313368		313368
*MOR*			1.12		1.12
*VPC*			7.75%		7.80%

The ever-adherent model (Table 2, model 2) was similar to the first invitation adherent model. Age showed the same U shape with lower adherence observed for the youngest and oldest screening age groups but in model 2, intermediate age showed lower odds ratios.

In the empty model including the health district instead of FDs practice, clustering by health district was also significant (LR test p = 0.023). However, in the multilevel model with fixed effects, HD explained almost no variability (VPC 0.006%) and was associated with a MOR as low as 1.002 (corresponding figures for the model including FDs practices were 4.8% and 1.11).

Thus the VPCs and MORs from multilevel models point to a moderate to important influence of FDs practice and a negligible influence of district on adherence.

**Fig 3:**
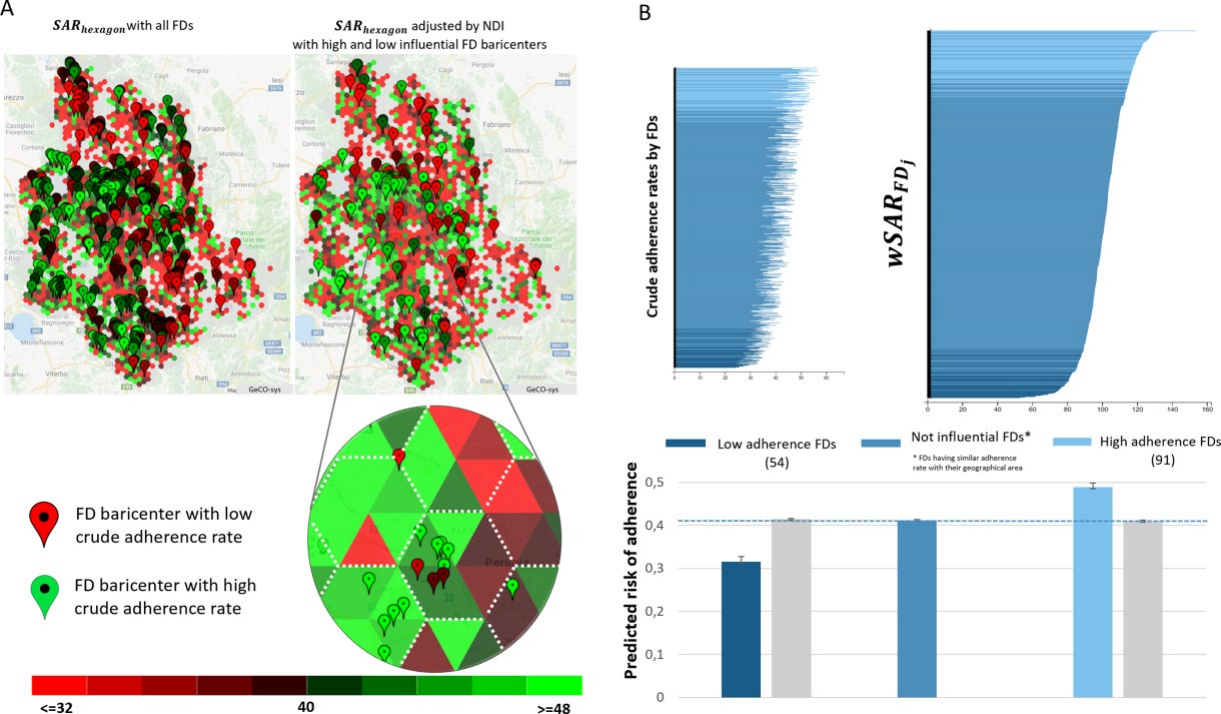
Risk of screening adherence by category of FD practices. A. FDs ${wSAR}_{S_{FD_{j}}}$ (the markers correspond to the practice baricenter and marker colors to adherence category) plotted against SARs for small triangular areas (about 0.50 km^2^). B. Top panel: crude adherence rates (left) and ${wSAR}_{S_{FD_{j}}}$ (right) by FD practices. Bottom panel: marginal predicted adherence probability for low adherence FDs and high participation FDs and, for comparison, adherence probability for a generic person at average level of covariates. Figure was created by the author Bianconi F. combing the caterpillar, bar plots and maps generate with GeCO-sys an extension of [21].

Locally weighted SARs comparing individuals in a FDs practice to residents in the same area are shown in Fig 3 panel A. In particular, we found that, respectively, 91 and 54 FD practices had local ${wSAR}_{S_{FD_{j}}}$ significantly higher or lower than the population living in the same areas (Fig 3 panel B). We compared adherence levels for high, average, and low FDs practice to further clarify the influence of FDs. Locally weighted SARs ranged from 51.4% to 152.4% and crude adherence probabilities from 21.0% to 57.0%. Foreign-born individuals were 5.0% among FD practices with low adherence and only 3.1% among practices with high adherence. However, the foreign-born showed a significantly higher adherence in the FD practices with high adherence than in the ones with low adherence (35.8 vs 25.7%, chi-square test p<0.0001).

Predicted adherence probabilities obtained from multilevel models including only significant FDs practices are illustrated in Fig 3 panel B. (see S1 Table for models). FDs practices with high level of adherence showed a 49% probability of adherence at first invitation whereas the corresponding Figure for FDs with low adherence was only 32%. To rule out the possibility that different adherence levels stemmed from clustering of individuals with unfavourable distribution of fixed-effect variables, we used the model including all invited people (model 1) to predict adherence for high, average, and low FDs practices. Indeed, the average predicted probabilities of adherence for individuals belonging to different FD practices was similar.

## Discussion

We found that FDs practices had a significant influence on colorectal cancer screening adherence in an organized screening setting. In terms of both explained variance and median odds ratio, the influence of FDs practice was important, after accounting for individual-level variables. In our study, clustering by FDs practice was associated with a magnitude of effect comparable to being in the most deprived group.

Population geocoding [21] allowed a new analysis comparing people in a FDs practice to people living in the same area. Three FDs groups were identified: a. n.91 (13.5%) physicians with patient participation significantly higher than people living in the same geographic area (“promoters”); b. n.54 (8%) physicians with significant low adherence (“opponents”) and c. the majority (n.531, 78.5%) of physicians, showing similar participation rates to the area population (“non-influential”). These findings provide additional evidence for the role of FDs behavior in determining CRC screening adherence. Based on models stratified by FDs group, we estimated that individuals in a promoter FDs practice had an adherence probability 17% higher than individuals in opponent FDs practices and 9% higher than the adherence probability for an invited person at average covariates values. The observed gap was not due to an imbalance in individual level covariates by FDs group (Fig 3).

Screening recommendation by FDs is a facilitator of participation [27,28]. Notably, in our study FDs were associated with different screening behaviours, even though they were involved in the organized screening program. Indeed, FDs signed the invitation letter [29], received a list of their patients non-attending screening or colonoscopy after a positive FIT result [30], and received financial incentives for high participation levels [31].

Further research will explore FDs clinical practice to identify activities and attitudes associated with successful or unsuccessful adherence rates. Diffidence toward cancer screening and/or the preference of screening modalities other than fecal testing could possibly explain the different FDs attitude [32–34].

The FDs perception of barriers to CRC screening participation results in significantly different FDs performances, as reported by Weiss et al. [35]. Barrier identification and perception may relate to active FD involvement in the screening campaign.

FDs association in mono- or multidisciplinary teams and their collaboration with health professionals (e.g., nurses) in promoting preventive interventions may have contributed to the observed variability and should be further investigated [36].

Clustering factors (e.g., selection of people with characteristic adherence rate in a practice) may also have contributed to our results. Indeed, the percentage of foreign-born patients in a FD practice was a significant clustering factor. The reduced screening participation could be due to linguistic or cultural barriers of a specific ethnic community, which could partially explain their tendency to group within the same FD practice [37]. Furthermore, a high percentage of foreign-born individuals in a FD practice could be associated with other established determinants of lower screening adherence (e.g., low educational levels, low income). Since FDs practices do have a geographic basis, the percentage of foreign-born individuals could be an indicator of neighbourhood deprivation, thereby linked to screening adherence [38]. Interestingly, the promoter FDs group had a low percentage of immigrants but with a relatively high screening adherence if compared to opponent FDs group.

The association between being born abroad and belonging to the most deprived quintile and to an opponent FD practice, resulted in a strikingly low screening participation (25.2%). Considering the low adherence to CRC screening registered in our study, foreign-born individuals represent a valid target for public intervention. Moreover, the relevance of immigrant participation will increase, as an increasing number of foreign-born people will match the age eligibility criteria for CRC screening in the near future. The percentage of invited foreigners was less than 6% in our study, but the percentage of residents born abroad in the pre-screening age (30–50 years old) was 20.8% in 2013 (data from the national institute of statistics ISTAT [39]).

With a much lower explained variance than FD practice, the local health district had almost no influence on screening adherence. This negative finding was surprising, as the HD is appointed to coordinate public health and FDs activity (particularly the team-based ones) and thus should play a relevant role in disease prevention.

Additional individual-level factors affected CRC screening participation, such as socioeconomic status, being born abroad and gender. People with a low SES level participated less in CRC screening. In our study, the decrease in participation rates started in the fourth quintile of deprivation and reached a probability as low as 39% among the most deprived. The impact of deprivation on CRC screening adherence has been described in several studies. In the UK, CRC screening uptake varied from 35% in the most deprived quintile to 61% in the least deprived quintile (overall participation 54%) [40] and Pornet et al. reported a similar gap for the most deprived [41]. In the French study, however, the least deprived participated in screening more than the intermediate socioeconomic status levels.

Being part of an ethnic minority and having a low income are significant barriers to screening participation in the majority of published studies [28].

Previous evidence showed that organized screening reduces the socioeconomic gradient in adherence to this preventive intervention, even though it does not eliminate the inequalities when compared to opportunistic screening [42]. Despite the availability of effective measures in a FDs practice which could improve screening adherence, tailored actions to reduce the impact of SES inequalities on participation should be further investigated [43]. Gupta et al. improved screening adherence through multilingual, low-literacy, educational brochures and reminder phone calls [44]. A similar intervention could be feasible and appropriate in our regional context.

In contrast with other studies [45], we found no effect of rural residence on screening participation, which is probably attributable to the minimal travel effort required by the test kit administration.

In our study, women were more likely to participate in the FIT-based CRC screening, in accordance with other studies [46] but more frequently the female gender represents a barrier to adherence [28]. The importance of participation in CRC screening among men is remarkable, since scientific evidence attributes the greatest benefit from CRC screening to males [47]. Age <65 years represented a barrier to screening participation in most studies [28]. In our study, age had a U-shaped influence on adherence. Reduced adherence in the youngest invited age group may depend either on an underestimation of CRC risk or on the perception of the screening invitation as a modern rite of passage into old age [48]. The oldest invited age group showed a reduced adherence in our study, despite the adoption of measures aimed to reduce geographical barriers and travel difficulties (e.g., mailed kit, test return by mail). No univocal result is reported in the published literature results for this age group [49,50].

Our study has limitations. Data on cluster lever covariates, which could explain variability by FDs practices, including FDs attitude about screening, were lacking. The SES indicator used in our study was available at census tract level (micro-ecologic) and not at an individual level. Moreover, the NDI index could have a reduced ability to measure socioeconomic status among immigrants [51].

## Conclusions

Adherence to CRC screening was low in our study. Thus, public health measures to improve participation in the regional population would be appropriate. In addition, targeted actions should be designed to increase screening adherence among males, the foreign-born and the most deprived. We showed that FDs practice influences screening participation by comparing adherence in a FD practice to that of people living in the same geographic area. In particular, “promoter” FDs practices with high adherence rates could provide effective models to improve screening participation.

## Supporting information

S1 Fig Regional screening adherence map of SAR_s_ by municipality and gender for all cases. The crude adherence probabilities are presented for overall dataset and first and third rounds (the yellow line is the average regional adherence). Figure was created by the author Bianconi F. combing the caterpillar plots and maps generate with GeCO-sys an extension of [21].
(TIF)

S1 TableEstimated odds ratios of adherence to CRC screening program with multilevel logistic regression models for FDs practices respectively with significantly higher (model 3) and lower (model 4) local **${wSAR}_{S_{FD}}$** than the residents in the same areas: Random variation of the intercept for FDs practices and the coefficient for the percentage of foreigners for adherents.(DOCX)
