# Supplementary figures and images for: Geospatial analysis of the influence of family doctor on colorectal cancer screening adherence
Source: PLoS One. 2019 Oct 4;14(10):e0222396. doi: 10.1371/journal.pone.0222396 (PMC6777754; doi:10.1371/journal.pone.0222396)

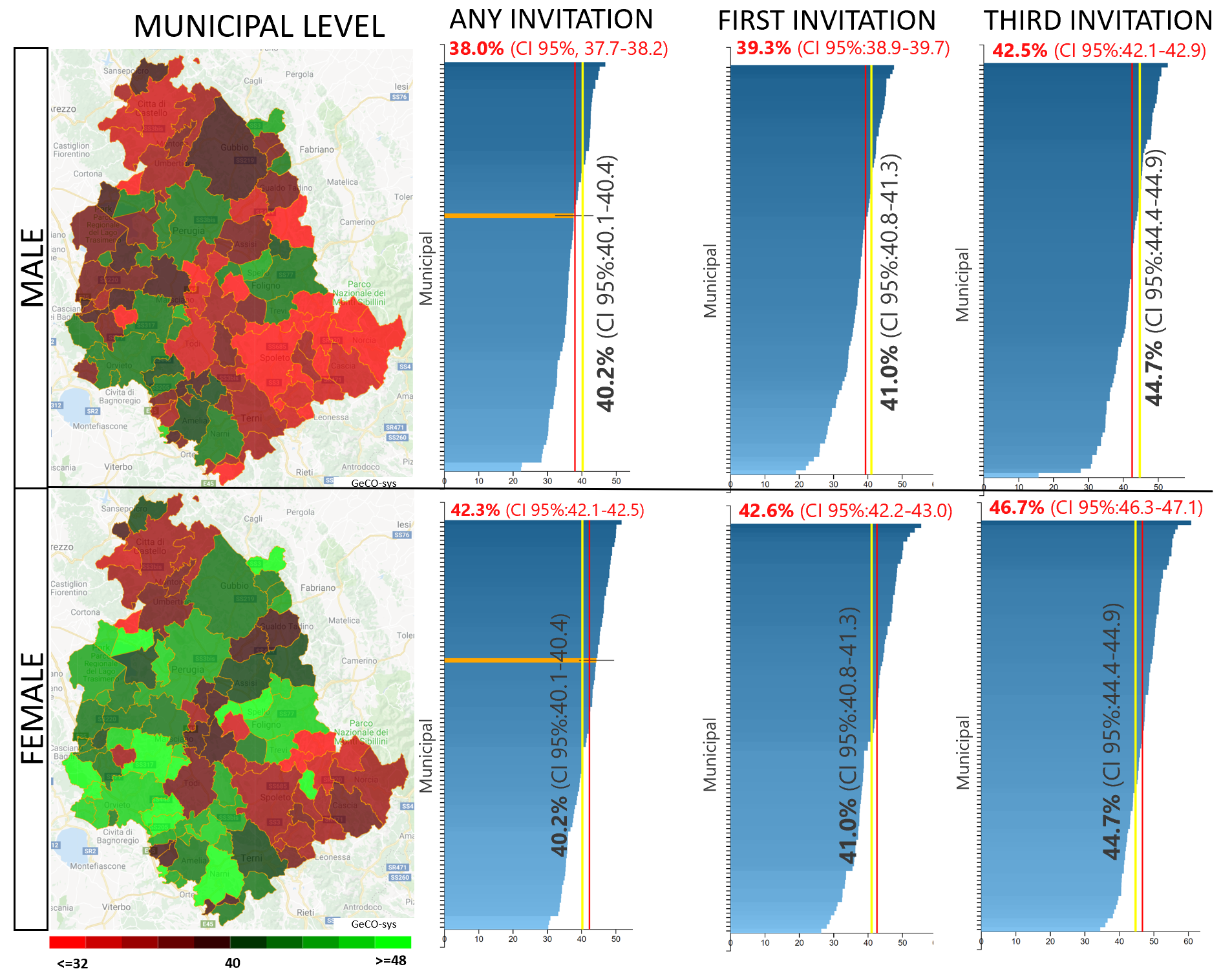

Supplement: S1 Fig — The crude adherence probabilities are presented for overall dataset and first and third rounds (the yellow line is the average regional adherence). Figure was created by the author Bianconi F. combing the caterpillar plots and maps generate with GeCO-sys an extension of [21]. (TIF) [file pone.0222396.s001.tif]
