# Supplementary material for: Geospatial analysis of the influence of family doctor on colorectal cancer screening adherence
Source: PLoS One. 2019 Oct 4;14(10):e0222396. doi: 10.1371/journal.pone.0222396 (PMC6777754; doi:10.1371/journal.pone.0222396)
Supplement: S1 Table — (DOCX) [file pone.0222396.s002.docx]

**S2 Table.** Estimated odds ratios of adherence to CRC screening program with multilevel logistic regression models for FDs practices respectively with significantly higher (model 3) and lower (model 4) local $\mathrm{wSAR}_{S_{FD}}$ than the residents in the same areas: random variation of the intercept for FDs practices and the coefficient for the percentage of foreigners for adherents

| **Variables** |  | **Model 3** | | **Model 4** | |
| --- | --- | --- | --- | --- | --- |
|  |  | **OR** | **95%CI** | **OR** | **95%CI** |
| *Sex* |  |  |  |  |  |
|  | *Female* | 1.21 | 1.17-1.26 | 1.20 | 1.14-1.27 |
|  | *Male (ref.)* | - | - | - | - |
| *Nationality* |  |  |  |  |  |
|  | *Italian* | 1.71 | 1.52-1.92 | 1.49 | 1.31-1.70 |
|  | *Other(ref.)* | - | - | - | - |
| *Socioeconomic Status (NDI)* |  |  |  |  |  |
|  | *1 Less deprived* | 1.09 | 1.03-1.15 | 1.12 | 1.03-1.22 |
|  | *2* | 1.09 | 1.02-1.15 | 1.03 | 0.95-1.12 |
|  | *3* | 1.08 | 1.02-1.15 | 0.94 | 0.86-1.02 |
|  | *4* | 1.08 | 1.01-1.15 | 1.01 | 0.92-1.10 |
|  | *5 Most deprived(ref.)* | - | - | - | - |
| *Age* |  | - | - | - | - |
|  | *50-54 (ref.)* | - | - | - | - |
|  | *55-59* | 1.20 | 1.13-1.26 | 1.17 | 1.07-1.27 |
|  | *60-64* | 1.39 | 1.32-1.47 | 1.27 | 1.15-1.39 |
|  | *65-69* | 1.47 | 1.40-1.56 | 1.24 | 1.12-1.36 |
|  | *70-74* | 1.09 | 1.03-1.15 | 1.19 | 1.08-1.30 |
| *Round* |  |  |  |  |  |
|  | *First (ref.)* | - | - | - | - |
|  | *Second* | 1.01 | 0.93-1.03 | 1.29 | 1.19-1.40 |
|  | *Third* | 1.01 | 0.93-1.04 | 1.39 | 1.25-1.56 |
| *N* |  |  | 49934 |  | 25038 |
| *MOR* |  |  | 1.13 |  | 1.10 |
| *VPC* |  |  | 7.85% |  | 7.00% |
